# African American Women: Surviving Breast Cancer Mortality against the Highest Odds

**DOI:** 10.3390/ijerph13010006

**Published:** 2015-12-22

**Authors:** Shelley White-Means, Muriel Rice, Jill Dapremont, Barbara Davis, Judy Martin

**Affiliations:** 1 Department of Clinical Pharmacy, University of Tennessee Health Science Center, 881 Madison, Suite 202, Memphis, TN 38163, USA; 2 Mustard Seed, Inc., 653 Mississippi Blvd, Memphis, TN 38126, USA; muriel_rice@att.net; 3 Loewenberg School of Nursing, University of Memphis, 3567 Community Health Building, Memphis, TN 38152, USA; jdaprmnt@memphis.edu; 4 Department of Management, Fogelman College of Business and Economics, University of Memphis, Memphis, TN 38152, USA; bddavis@memphis.edu; 5 Shelby County Health Department, Memphis, TN 38105, USA; judy.martin@shelbycountytn.gov

**Keywords:** breast cancer, African American women, health disparities, survivorship, patient/provider communication, focus group

## Abstract

Among the country’s 25 largest cities, the breast cancer mortality disparity is highest in Memphis, Tennessee, where African American women are twice as likely to die from breast cancer as White women. This qualitative study of African-American breast cancer survivors explores experiences during and post treatment that contributed to their beating the high odds of mortality. Using a semi-structured interview guide, a focus group session was held in 2012 with 10 breast cancer survivors. Thematic analysis and a deductive *a priori* template of codes were used to analyze the data. Five main themes were identified: family history, breast/body awareness and preparedness to manage a breast cancer event, diagnosis experience and reaction to the diagnosis, family reactions, and impact on life. Prayer and family support were central to coping, and survivors voiced a cultural acceptance of racial disparities in health outcomes. They reported lack of provider sensitivity regarding pain, financial difficulties, negative responses from family/friends, and resiliency strategies for coping with physical and mental limitations. Our research suggested that a patient-centered approach of demystifying breast cancer (both in patient-provider communication and in community settings) would impact how women cope with breast cancer and respond to information about its diagnosis.

## 1. Introduction

Breast cancer in the United States is the most common cancer in women ages 45 through 64. According to the American Cancer Society, in 2014, about 232,670 women were diagnosed with breast cancer, and almost 40,000 of them died of the disease. It has been confirmed that race plays a role in breast cancer incidence and survival. While White women are more likely to get breast cancer, African American women are more likely to die from breast cancer than any other group [1]. The five-year survival rate for breast cancer is 90% among White women and 79% for African American women [1]. The costs of breast cancer treatment are non-trivial and thus its differential experience by race is worthy of investigation. Over a five-year treatment period, the societal cost of metastatic breast cancer is $12.2 billion or $98,571 per patient per year [2].

Compared with the country’s 25 largest cities, the breast cancer mortality disparity is highest in Memphis, Tennessee, where an African American woman is 2.09 times more likely to die from breast cancer than a White woman [3]. The national mortality rate ratio of breast cancer deaths for African American women compared to White women is 1.4. Why is Memphis the city with the highest racial disparity in breast cancer mortality? Whitman, Orsi, and Hurlbert (2012) noted two critical factors associated with higher mortality rate ratios are low median household income and high segregation, suggesting that financial and geographical barriers to care may be critical [3]. Demographic data for Memphis are consistent with this research hypothesis that there is a high correlation between breast cancer mortality disparities, median household income, and segregation.

In comparison to US national census reports, where median household income was $51,017 and 15% of US residents lived in poverty, in Memphis, median household income was $37,072 and 25% of residents lived in poverty, in 2012 [4,5]. Additionally, the index of dissimilarity in Memphis, regarding segregation, is 72.2, indicating that the city has a very high level of racial segregation and over 72 percent of Memphis’ White residents would need to move to neighborhoods more heavily populated by African Americans in order for Whites and African Americans to be evenly distributed across all neighborhoods [6]. 

Although African American women face a high relative risk of breast cancer death in Memphis, some African American women beat the odds. What is the story behind their survival? 

This article reports the results of a qualitative study of African American breast cancer survivors in Memphis, Tennessee. Despite over a decade of having the highest breast cancer mortality disparity among the largest metropolitan areas of the country, this study provides the first in-depth qualitative exploration of the insights of African American breast cancer survivors in Memphis. Our objectives were: (1) to describe the breast cancer experiences among African American women in Memphis, and (2) discover resources African American breast cancer survivors stated contributed to surviving breast cancer.

### 1.1. What We Have Learned about the Breast Cancer Experience of African American Women in Other Parts of the Country

Recent literature on breast cancer experiences of African American women has focused on psychosocial, economic, and cultural influences. Many researchers report that spirituality is central in the coping process [7,8,9,10,11]. Others identify family support as critical [11]. Barriers and concerns identified in the literature for African American women include perceptions of inadequate support and lack of information from doctors, insurance restrictions, and lack of knowledge [7,9,10,11,12,13].

For many African American women, having a relationship with God helped them to believe that they could survive cancer, and that God would bless them with prolonged life [7]. Spirituality helped survivors to have a positive attitude and classify themselves as “healed” [6]. God provided support that they could not get from their family and friends [14]. Surviving breast cancer even enhanced spirituality and the prayer lives of survivors increased [9].

Depression was a common occurrence, with many women feeling guilty that they had survived and others had not [7]. Another emotional concern was the fear of reoccurrence [7]. Other psychosocial concerns were worries about children and not wanting to be a burden on family members [11]. Survivors noted that a caring and loving family was essential for their positive prognosis [15]. 

Among African American women, patient trust in physician interactions and a belief that providers will take their health concerns seriously was central to preventing diagnostic delays between the time that a breast abnormality is identified and a biopsy or surgery occurs [12]. Darby, Davis, Likes, & Bell (2009) noted that insurance limitations results in missed, delayed, or fewer treatment opportunities [13]. Mollica and Nemeth [7] reported that African American breast cancer survivors do not feel adequately prepared for the financial, social, and stigma challenges experienced during as well as after cancer treatment [7]. The impact of chemobrain on cognitive function has been one of the unexpected surprises during post cancer treatment [16]. Personal crises were perceived by survivors if breast cancer was considered a phenomenon that impacted beauty, future relationships with men, or the ability to marry and have children [10]. Aziz and Rowland (2002) emphasized that to better understand the survival of ethnoculturally diverse and medically underserved survivors, it was important to assess the impact of the health care delivery system and cultural acceptance of care [17]. 

Using the social-ecological framework, health disparities researchers emphasized that health promotion occurs at five levels: individual, interpersonal, institutional, community, and public policy [18]. Most of what we know about the breast cancer experiences of African American women as espoused through their voices via qualitative research reflected their perceptions of how the lower three levels of influence (individual, interpersonal, institutional) have impacted their survivorship. Quantitative research has also captured a role of community/neighborhood in influencing diagnostic follow-up from mammography screenings [19]. This research indicated that racial segregation and its associated racial disparities in access to mammography screening, as well as living in neighborhoods with intense poverty and high levels of neighborhood crime, positively affected late diagnosis of breast cancer and negatively affected mammography screening and regular adherence to mammography screening guidelines. Similar to African-American women in other parts of the country, when asked to share insights about their breast cancer experience, women in this study primarily focused on the three lower levels of factors that influence health outcomes. However, the African American women in this study resided in a city with a high risk of exposure to negative social determinants of health that could adversely impact their breast health. Given this social/community context and using qualitative analysis, we explored themes that are important in understanding their survival, comparing these themes with those of African American women nationally whose social/community environments were very different from that of Memphis, Tennessee.

## 2. Methods 

### 2.1. Design and Setting

This focus group study that used descriptive qualitative analysis surveyed female African-American breast cancer survivors who were part of breast cancer support groups in 2012 in Memphis, Tennessee, one of the largest cities in the Mid-South region of the United States. A purposive sampling strategy was used. Ten participants were part of the focus group, which was conducted by an experienced focus group facilitator. The focus group lasted about 90 min. During data collection, participants demonstrated a variety of emotions and at times cried, laughed, joked, and even became angry as they discussed their experiences. Interviews were transcribed by an experienced transcriptionist and reviewed by the interviewer and researcher for accuracy of transcription. The rationale for using a focus group was to learn a great deal about what African American breast cancer survivors perceived, remembered, and described as their experiences as they progressed from diagnosis to treatment to remission. Focus groups yielded information from individuals about their meanings, interactions, and the interpretative process used by the individual dealing with the experience. This type of yield was what Patton (2002, p. 112) identified as the strength of this strategy [20].

### 2.2. Instruments

**Figure 1:**
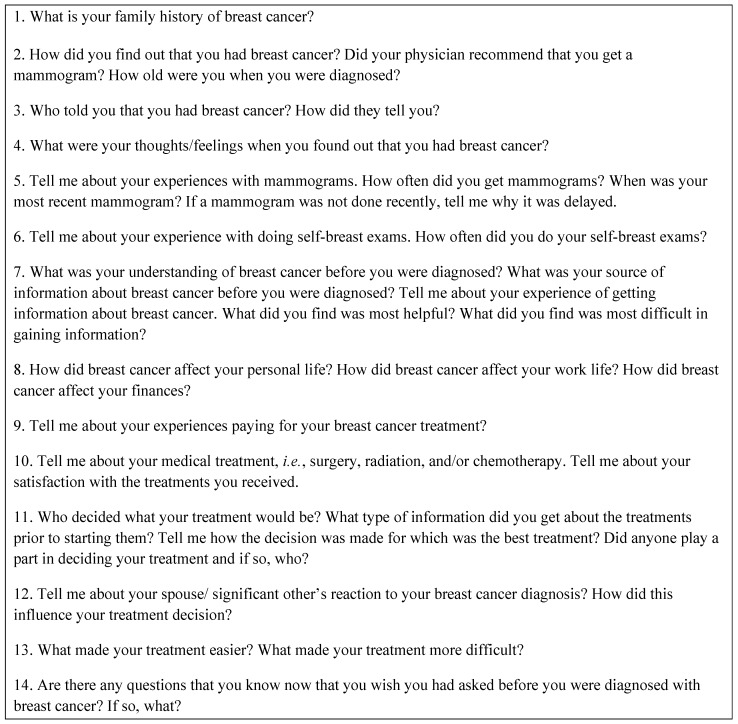
CHEER * study of black women who survived breast cancer: semi-structured interview guide. (* CHEER is the acronym for Consortium on Health Education, Economic Empowerment and Research.)

A fourteen question semi-structured interview guide (Figure 1) was developed by the investigator and reviewed for appropriateness to the topic by two outside experts to promote content validity and establish rigor and trustworthiness. Questions were developed using insights gleaned from the Metropolitan Chicago Breast Cancer Task Force’s town hall meetings that elucidated and illuminated breast cancer concerns in another area of the country with high breast cancer mortality disparities [21]; Chicago is ranked fifth nationally in racial disparities in breast cancer mortality [3].

### 2.3. Procedures: Data Collection

Institutional review board approval was granted for this study from the affiliated university. The researcher solicited two groups; an African American women breast cancer support group and an African American church-based breast cancer support group. Individuals who agreed to participate were given the date and location of the focus group. After individuals presented for the focus group, written consents were obtained from individuals who agreed to participate in the study. Next, the focus group was lead with all participants who presented to the session and signed written consents. Participants were encouraged to be open and honest and were asked open-ended questions followed by probing questions when necessary to reveal details regarding reported experiences. Fourteen questions were asked using the researcher developed questionnaire guide below (Figure 1) and follow-up probe questions were used. Lastly, individuals were given a Kroger gift card in the amount of twenty-five dollars ($25) for participating in the focus group. 

### 2.4. Data Analysis Strategy

Qualitative data analysis techniques were used to illuminate the data. Open coding of the data began with the two qualitative researchers reading the transcripts of the interviews, and then manually identifying phrases and words. During open coding, phrases and words were highlighted and circled that described behavior of the survivors. Data management was performed through development of suitable classification or coding schemes, by breaking down the data into discrete parts, closely examined, paralleled for similarities and differences and compared further in discussions. A thematic content analysis and deductive a priori template of codes were used to make sense of the data [20,22,23]. This method produced a structure to handle, organize, and derive meaning from the focus group data. The template for the coding was based on the interview questions. Internal quality and trustworthiness were attained by having two researchers (both authors on the paper) independently code the data and form consensus through analysis. The data analysis reported in Table 1 and Table 2 represented demographic characteristics and themes and sub themes deducted from an analysis of discussions during a focus group of breast cancer survivors. 

## 3. Results

Table 1 reports the demographic characteristics of the ten breast cancer survivors who participated in the focus group. They were all African American women who were members of local breast cancer support groups and who ranged in age from 38 to 62. The women represented diverse socioeconomic backgrounds, including education ranging from high school graduates to Ph.D.s. Some were single or divorced, and others were married.

**Table 1:** Demographics of African American breast cancer survivors for Consortium on Health Education, Economic Empowerment and Research (CHEER) focus group.

Demographics	
Number of Participants	10
Gender	Female
Age Range	38–62
Ethnicity	African American
Family History	7 had family history of breast cancer

Table 2 (column 2) reports the five major themes that were identified in the focus group: (1) Family History of Breast Cancer, (2) Breast/Body Awareness and Preparedness to Manage a Breast Cancer Event, (3) Diagnosis Experience and Reaction to the Diagnosis, (4) Family Reactions, and (5) Impact on Life. Themes were extracted from survivor responses to several of the questions that were listed in Figure 1. Thus, in column 3 we reported subthemes that were numbered according to how they corresponded with survey questions reported in Figure 1. To identify a distinct individual response, a subtheme in column 3 included a number after the decimal point. For example, a response in the subtheme column was numbered 2.1.2; this is person number one’s second response for the subtheme, “Knowledge of Physical Changes prior to breast cancer diagnosis,” that was obtained by asking her question number 2 from Figure 1. A summary of participants’ responses was organized according to the five major themes. 

**Table 2:** Five major themes of African American breast cancer survivors for CHEER focus group.

Theme ^#^	Theme	Sub Themes (with Applicable Quotes)	Quotes Related to Sub Themes
1	Breast Cancer in the family	1.1 First in family to have it 1.2 No one in family wanted to be genetically tested 1.3 other relatives had it	
2	Breast/body Awareness and Preparedness to Manage a Breast Cancer Event	Knowledge of Physical Changes prior to breast cancer diagnosis 2.1.1 lump 2.1.2 nipple “turned under” 2.3 hair falling out and unable to take perm 2.4 losing weight 2.5 Low vitamin D level 2.6 Lymph nodes swollen	
		Information source of breast cancer prior to diagnosis 7.1 no one 7.2 clinic/doctor office 7.3 co-workers 7.4 sister in medical school had some information	7.1 “Black people don’t do preventive measures until it hits us.” 7.3 “A co-worker in late 20s had prophylactic double mastectomy but she was White so it didn’t sink in.”
		Regular Mammogram 5 went yearly	“Skipped the 3rd year and 4th year lump found”
		Self-Breast Exams 1	“Tried but didn’t know what I was feeling” “Did haphazardly but unsure” “Have implants and nerve damage from surgery so I’m afraid I wouldn’t feel it”.
		Preparedness to follow-up regarding mammogram results	“..found lump in 99. The doctor wanted to watch it. …returned in 2003 in stage 2...” “Yes I ignored the results & I went back; it was a stage 3” “…didn’t listen about my lymph nodes. No lump but swollen lymph nodes…. they kept saying I had an infection from my hysterectomy but my GYN followed-up…”
3	Being Diagnosed and Reacting to the Diagnosis	Informer of the Diagnosis 3.1 surgeon 3.2 nurse 3.3 sent a letter 3.4 phone call at work 3.5 had to demand I be told 3.6 technician	3.5 “Dr. had ulterior motives. He wanted me in his friend’s clinical trial….”
		Emotions on hearing the diagnosis 4.1 numb 4.2 disappointed 4.3 disbelief 4.4 cried 4.5 attack mode	4.5 “I thought it was an old people disease.”
		Behaviors after hearing news 4.1a text everyone 4.2a prayed 4.3a went back to work as soon as possible	
4	Family Reaction to Breast Cancer Diagnosis	4.1b friends seemed to feel betrayed because I wasn’t emotional 4.2b brother thought I was crazy for trusting the doctors 4.3b people gave me a hard time 4.4b my children started acting out at school 4.5b co-worker said stop crying and get a mastectomy	
5	Impact of Breast Cancer Treatment on Life	Things that made treatment difficult 13.1a couldn’t stomach food 13.2a couldn’t eat or drink 13.3a doctor not believing I was in pain after surgery 13.4a didn’t take pain meds because scared of the side effects. 13.5a hot flashes	13.3a “After all of that surgery… and they gave me Ativan, instead of pain pill, I felt like that was just… I was resentful toward them [doctors] because of that”. 13.4a “… they tell you about the side effects…so I didn’t take any pain pills… I thought the pain was because of the surgery… I’m not use to taking drugs… after 9 months I said something I was in so much pain”.
		Things that made treatment easier 13.1 prayer 13.2 following directions 13.3 family support 13.4 chores shared	
		Spouse/significant other response to the diagnosis 12.1 husband said it didn’t matter to him 12.2 It didn’t matter what they thought—it was my body 12.3 boyfriend left	12.3 “My gynecologist said to me, don’t do if for the man. You know Black men ‘gonna’ sneak around anyway. So don’t be surprised at by the end of this journey, you are by yourself”.
		Cancer’s impact on finances 8.1 had to retire (×3) 8.2 had to work two jobs 8.3 got jobs that didn’t require as much stress or responsibility	
		Paying for treatment 9.1 clinical trial 9.2 insurance (×2) with out of pocket expenses 9.3 got attorney (2) 9.4 social security	9.3 “I’m an in-between” can’t get Medicare or Social Security. 9.4 “… Social Security Administration won’t grant benefits unless the cancer has metastasized because they said a survey of women with breast cancer states it won’t last more than a year”.
		Things I wished I knew to ask about 14.1 harvesting my eggs in the event I get married 14.2 effect of chemo on sexual drive and desire 14.3 types of breast cancer & treatments for each type 14.4 types that affect African Americans 14.5 more details and warnings	14.2 “being so young I was at the top of my sexual peak… after chemo… I went straight to menopause”. 14.4 “I would have been more proactive about my health”.

Note: Numbers for subthemes correspond with interview guide questions. Numbers after decimal point identify unique individuals.

### 3.1. Family History of Breast Cancer

Breast cancer was indeed a part of the family history of survivors, reflecting prior knowledge about the breast cancer experience. Two survivors (20%) were the first in their family to have breast cancer. Of those with a family history of breast cancer, two reported that it was their mother who had experienced breast cancer. Most talked about aunts who had experienced breast cancer. One woman sought genetic testing and found that breast cancer was not hereditary in her family.

### 3.2. Breast/Body Awareness and Preparedness to Manage a Breast Cancer Event

The survivors were aware of the physical changes and symptoms indicating a potential problem of breast cancer. Survivors indicated the presence of lumps, a nipple “turned under,” “hair falling out” and “inability to tolerate a perm,” “losing weight,” “low vitamin D levels,” and “swollen lymph nodes.”

Monthly breast self-exams were not a routine part of any of the survivors’ preventive health strategies. Prior to surgery, survivors indicated that they had been unsure of the correct technique for performing a breast self-exam, and those who attempted the self-exam stated it was done haphazardly. Post-surgery and treatment, all survivors now perform a monthly breast exam, with one survivor examining her breasts every time she takes a shower. Most of the survivors (70%) received a regular mammogram prior to diagnosis. Currently, survivors participate in a regular regimen of mammograms and have become better prepared to identify any breast cancer concerns. 

Because many survivors were not mentally prepared to manage a breast cancer event, they did not immediately seek follow-up treatment once breast cancer was discovered. The following examples illustrate this conclusion.

“I found a lump in 1999. The doctor wanted to watch it. I returned in 2003 in stage 2 breast cancer.”

“…I ignored the results and I went back; it was stage 3.”

“I didn’t listen about my lymph nodes. (Doctors) kept saying I had an infection from my hysterectomy, but my gynecologist followed up and ……it was breast cancer.”

Before diagnosis, some survivors (4) had not received information about breast cancer. For those who had, information was available from clinics and doctors’ offices, co-workers, friends, and family. The quotes below illustrate the survivor’s perceptions of the racial/cultural difference in breast cancer preparedness between them and White women.

“Black people don’t do preventive measures until it hits us.”

“A co-worker in her late 20’s had prophylactic double mastectomy, but she was White so it didn’t sink in.”

### 3.3. Diagnosis Experience and Reaction to the Diagnosis 

Both direct and indirect communication strategies were used to inform the survivors about their positive diagnosis of breast cancer. Some survivors heard from the surgeon, one heard from a nurse who prepared her for the unexpected when the doctor called the next day, others were sent letters in the mail, one received a phone call at work, and for another she heard nurses whispering in the background and then a radiologist informed her. One survivor indicated her skepticism about the information provided by the provider because she thought the “Dr. had ulterior motives. (The doctor) wanted me in his friend’s clinical trial. The surgeon took a needle biopsy that he told me would be definitive, but he wanted to do another test after the results came back. He said the first test was not definitive. I said I would not have taken a test that was not definitive, so I said no to additional tests and stated, ‘Just tell me.’ He then said yes, it is breast cancer.” 

The reported emotion experienced most by survivors (7) after hearing of the diagnosis was that of feeling numb. Other responses were disappointment, disbelief, and crying. Some survivors relied on their faith using strategies such as: (1) praying that the Lord would take care of this, (2) remembering that every journey is a blessing, and (3) going home and writing and placing in a drawer a note that said, “I have cancer.” Next the survivor prayed to God and said, “Let Your will be done.” Concerns for family members were also immediate reactions; one survivor was concerned about her young kids, another was concerned about a husband who had just had a stroke, and there were concerns about how kids, sisters and brothers would cope. 

The youngest survivor indicated that she went into attack mode; she thought breast cancer was an old person’s disease. She stated:

*“I called it the attack mode. I was like, ‘What can we do, what do we have to do? How soon can we get it out? I want everything done now.’ If I could’ve had my surgery the next day, I would have. I didn’t want to wait for anything. You know. As soon as you can get it done, as soon as we could get it out, as soon as I could get through chemo. My mind was like as soon as I can get everything done, I can be done with this. But I never cried.”*

Another reaction by one survivor who felt numb was to send text messages to everyone saying that it was cancer. The message was: They said it’s cancer. After the mass email, the text recipients started calling her and her phone was “ringing off the hook.” A numb survivor coped by going back to work two days later. Her co-workers wanted to know why she was at work and she responded:

“I’m like, we going to work this out. They going to do this on my off days and in between. And I’m just going to keep going on and on and on.”

### 3.4. Family Reactions to the Breast Cancer Diagnosis 

Not only were survivors affected by the breast cancer diagnosis, but family members were also affected. The following depicts family reactions:

*“Friends seemed to feel betrayed because I wasn’t emotional.”*

*“My brother thought I was crazy for trusting the doctors.”*

*“Some people gave me a hard time.”*

*“My children started acting out at school.”*

Family and friends appeared to want the survivor to respond in the same way that they would to the receipt of breast cancer diagnosis, and were disappointed if that did not occur. Children feeling the need to be supportive of their mother at home released their frustrations in negative ways at school. 

### 3.5. Impacts of Breast Cancer Treatment on My Life 

Survivors found the breast cancer treatment process a challenge for their lives. Prayer, following provider recommendations, family support, sharing of chores, and affiliation with a support group made the treatment process easier. However, experiencing inability to eat or drink and hot flashes made treatment difficult. Patient-provider communication and provider responses to patients’ needs complicated the treatment process. For example, survivors stated concerns about not obtaining adequate pain relief, and a lack of communication from the doctor about the physical impacts of treatment:

*“My most difficult part was when my doctors didn’t believe that I was in as much pain as I was. After all of that surgery that I had and they gave me Ativan instead of pain pills. And I felt like that was just… I was resentful toward them because of that.”*

*“My thing was, they tell you about the side effects and everything, like I said, I wasn’t prepared for it. So I didn’t take any pain pills. Even though I was hurting for the first nine months I just thought it was a side effect. I was hurting bad. I be drawed up in the fetal position. But I didn’t know until one day I said something to the oncologist. He said, why you never said anything. I just thought, y’all keep saying it’s going to be so many side effects so just thought this was part of it and its going to go away. And they could not believe that all that time I had been doing chemo I never said anything. They was like, why… I just said, I thought it was a side effect. And I’m not used to taking drugs, I just thought that eventually. But it did. And it still hasn’t gone away. That’s when they sent me to a pain management specialist. To help me.”*

*“The doctor didn’t inform me about how much time it was going to take to complete the treatment process and how physically debilitating it would be.”*

*“I was uninformed about the reconstruction procedure. No one shared information about physical changes after the operation. I woke up from surgery and felt butchered.”*

Survivors’ family lives were also impacted by breast cancer treatment. In one case, one survivor’s children had to “go live with other family members.” While one survivor stated it did not matter what her family thought because it was her body, others were concerned. One survivor who experienced her third round of breast cancer, was thankful that her husband had taken care of her and did not leave her. Another survivor’s boyfriend left her:

*“My gynecologist said to me, don’t do it for the man. You know black men ‘gonna’ sneak around anyway. So don’t be surprised at the end of this journey, you are by yourself.”*

There were physical and mental health impacts on the survivors. They reported depression, less energy, that it was hard to hold on to things (paresis), bone pain, vitamin deficiency, decreased to absent sex drive during chemotherapy, destroyed intestines, and newly developed arthritis. Experiences of having chemobrain were reported and survivors had problems with multi-tasking and remembering, as well as the development of coping strategies such as writing things down and using a blackberry. One survivor who experienced physical fatigue coped by replacing going to the gym with walking and opening her eyes to the things that she could do. 

A common experience was a change in work life. Three of the survivors retired. One had to work two jobs in order to manage. Another changed jobs, moving from one that required use of skills in processing numbers to a job that required use of less cognitive skills. 

Financial strains were also experienced. Most of the survivors had insurance, with two reporting excessive out of pocket expenses via their co-payments. One participated in a clinical trial. Transitioning between insurance/reimbursement providers (job insurance *vs.* TennCare (Tennessee’s Medicaid) *vs.* Social Security) was noted as also causing challenges with payments. One survivor noted:

*“…Social Security Administration won’t grant benefits unless the cancer has metastasized because they said a survey of women with breast cancer states it won’t last more than a year.”*

A survivor who described herself as an ‘in-between’ and not yet able to get Medicare or Social Security reported that she had to get an attorney in order to address payment for her treatment. 

In considering the statement, “Had I known, this is what I would have asked about before I was diagnosed with breast cancer,” survivors stated:

- Harvesting my eggs in the event that I got married
- How chemo impacts the body and all the problems I have now
- Effect of chemo on sexual drive and desire. “Being so young I was at the top of my sexual peak…after chemo…I went straight to menopause.”
- Types of breast cancer and treatments for each type
- Types of breast cancer that affect African Americans. “I would have been more proactive about my health.”
- More details and warnings

Every survivor agreed that their lives had changed forever, due to breast cancer. They now appreciate life a whole lot more. 

## 4. Discussion

Interestingly, concerns that were important to African American women in Memphis were similar to those of African American women nationally [5,9]. Prayer, family, and survivor support were central to coping with breast cancer. Lack of provider sensitivity to survivors’ concerns about pain was a repeated complaint. Difficulty in meeting financial responsibilities and lack of sexual desire were also reported. 

Unique concerns among African American breast cancer survivors in Memphis were cultural acceptance of racial disparities in health outcomes, reporting of the negative repercussions survivors experienced from family and friends, coping strategies to counter physical and mental limitations resulting from breast cancer, and payment issues associated with Medicare, Social Security, and legal recourses. 

The cultural acceptance of racial disparities in health outcomes was surprising. The researchers did not anticipate that African American women perceived that there were differences between themselves and White women in their attitudes about prevention, and that they thought these differences were culturally acceptable. It was also clear that these breast cancer survivors perceived that there are physiological differences in the experience of disease between White and African American women, so much so that one survivor discounted the knowledge shared with her by a White co-worker. She did not perceive that the life experience of a White woman could inform her about her health outcomes. Our review of the literature did not identify this type of perception of cultural differences by breast cancer survivors. 

Family history of breast cancer was the case for 7 of 10 participants of the focus group. The researchers speculated that part of the reason that these women survived was because they knew beforehand that breast cancer was a possibility for their lives, given a family history of the condition. Thus, prolonged denial that they were experiencing breast cancer was less likely than for women who were the first person in their families to experience breast cancer. In addition, they had information about what potential breast cancer outcomes could be for someone with their family history, *i.e.*, death or survival. 

While we speculated that one reason these women were survivors was because they had been vigilant in their preventive health practices prior to diagnosis, this was not confirmed by focus group participants. Their prevention strategy relied solely on technology, the annual mammogram. Breast self-exams were not routinely practiced and the survivors lacked knowledge about how to properly conduct a breast self-exam. As a result of lack of knowledge concerning breast self-exams, early detections of breast cancer were possibly delayed. Fortunately, these survivors did not lack access to technology via insurance coverage. This may not be the case for many low income women living in Memphis because funds for subsidized mammogram programs have not been sufficient to address the needs of this population; therefore, many low income women have not gotten timely screenings resulting in more late stage diagnoses.

The revelation that survivors were not always vigilant in seeking treatment after diagnosis was troublesome. Survivors reported delays in seeking treatment that lasted for years, resulting in advanced stages of breast cancer at the time of treatment. A better understanding of the causes of these delays could provide insights on how to improve outcomes among women who have been diagnosed but never followed up with necessary treatments that could save their lives. Maly *et al.* [12], suggested that one primary reason for these delays may be patient self-efficacy, *i.e.*, their confidence in their ability to get needed information and attention from their primary care provider. It was also important to understand what factors motivated these women, after years of delay, to finally make the decision to move forward in their treatment process.

Data confirmed that survivors were mostly insured, but with experiences of the insured varying. Insurance carriers forestalled some economic hardships for these survivors and gave them choices in the use of treatment services. Co-payments and other out-of-pocket expenditures varied and the degree of financial hardship experienced by those facing these expenses depended on the income resources available to the women. Survivors informed researchers that they transitioned from employed to retired/unemployed resulting from experiences with breast cancer and its treatment. Thus, health insurance status was not always static, with some women transitioning from employer insured to uninsured to state government/TennCare insured to disability coverage under Social Security. Transitions in insurance coverage cycles suggested varying levels of financial hardship throughout the experience of breast cancer, and potential variations in treatment decisions impacting survivorship. Darby *et al.* [13] similarly reported extreme financial hardship among breast cancer survivors in Tennessee and emphasize the importance of providing care across the continuum to address the complex needs of low-income cancer survivors.

Survivors had both positive and negative perceptions of breast cancer treatment. There were challenges in patient-provider communication and in provider efforts to prescribe adequate pain medications. Too little information was shared between providers and survivors about what patients should expect during and after treatment. Nonetheless, similar to many African American survivors in other parts of the country, prayer and family support were essential for the treatment process [6,7,9,14]. Implementing instructions, sharing of chores, and survivor support also made the treatment process easier. 

Survivors also showed an amazing resiliency, after they were able to recover from numbness. They learned to modify exercise routines in ways that fit their abilities. They changed jobs in order to maintain their earnings potential, constrained by the physical limitations that the aftermath of breast cancer treatment imposed. They made the choice to live with the aid of breast cancer treatment, even if that choice meant that they would lose their significant other. 

African American breast cancer survivors can serve as key informants for other women and health care providers. Survivors looked back retrospectively and shared what they wished they had known, wished they had done differently, thought had helped them to have a positive experience, learned as they researched breast cancer, and considered to reach the conclusion that breast cancer should not be feared. Thus, they are valuable community resources.

Our research suggested that a patient-centered approach of demystifying breast cancer (both in patient-provider communication and in community settings) would have an impact on how women cope with breast cancer and respond as recipients of information about its diagnosis. While many interventions to reduce disparities in breast cancer mortality have focused on getting women initial screening exams, another area of focus for survivors was strategies for surviving breast cancer. A clear message from the survivor participants in this study was that a focus on successful survivorship is also warranted.

This research also informed patient-centered interventions designed to improve breast cancer disparities outcomes. Mollica and Nemeth [7] noted that culturally competent interventions should be tailored to the community setting in which survivors reside. Thus, the findings of this research suggested that patient centered breast cancer care should focus on what patients identified as gaps in their care, e.g., information on the physical challenges faced post treatment, how to harvest eggs, types of breast cancers that affect African American women and treatments for each, symptoms, and impact on sex drive. 

## 5. Study Limitations

The participants in this study were African American breast cancer survivors in Memphis, Tennessee and their perspectives may not be generalizable to the total population of breast cancer survivors. Further, while we had representation of women from diverse socioeconomic backgrounds, their demographic distribution was not the same as that of Memphis breast cancer survivors and overrepresented some groups and underrepresented others. In addition, we did not obtain information on stage of breast cancer at diagnosis, which is important information to include in the demographic table, given the substantive concerns raised in the literature about relatively late stage of presentation among African American women. 

## 6. Conclusions

This qualitative study set out to explore the experiences of African American breast cancer survivors who live in a city with the highest odds of racial disparities in breast cancer mortality. The relevance of the findings can be used to educate African American women who might be impacted by breast cancer. In addition, healthcare providers can learn from the experiences of the survivors in this study and offer further education and supportive information for those experiencing breast cancer. 

Further study of the effectiveness of communication between breast cancer patients and their providers is needed. Survivors indicated challenges in communicating with providers such as (1) lack of provider sensitivity about pain, (2) feelings that providers had ulterior motives, and (3) concerns that the provider did not disclose complete information about the treatment process and how debilitating it is. These communication challenges surely limit the ability of patients to perceive that their providers are their advocates. In this regard, future research should survey providers to obtain a clearer understanding of what they are telling African American women. Additionally, future research should explore the effectiveness of communication within African American breast cancer support groups in order to identify how these groups fill the communication gaps between patients and providers and how communication within support groups impacts positive breast health outcomes. 

Given the substantial, and potentially negative, role of African American men in women’s experience of breast cancer treatment and survivorship, it is also important to understand their perceptions of the impact of breast cancer on womanhood. Perhaps interventions may need to focus on caregiver/partner supportive care for survivors. 

Our qualitative data was rich in its ability to help us glean important themes at the individual, interpersonal, and institutional levels that are associated with surviving a high risk of breast cancer mortality. Nonetheless, a population based study of a larger sample of African American breast cancer survivors in Memphis, and associated quantitative analysis, will facilitate an understanding of how these themes vary by socio-demographic characteristics of the survivors, as well as by the social/contextual characteristics of the neighborhoods in which they reside. For example, additional insights could be gleaned about patient-provider communication and how its effectiveness varies by the patient’s educational background. Other areas of exploration include a longitudinal perspective on the patient’s transitions between rounds of breast cancer incidence, transitions in employment during and after breast cancer treatment, and associated transitions in health insurance coverage and financial resources over time. Finally, it would be possible to explore the interactive effects of community level factors with individual, interpersonal, and institutional level factors.
